# Supplementary material for: A Simple and Rapid Protocol for the Isolation of Murine Bone Marrow Suitable for the Differentiation of Dendritic Cells
Source: Methods Protoc. 2024 Feb 27;7(2):20. doi: 10.3390/mps7020020 (PMC10961764; doi:10.3390/mps7020020)
Supplement: Supplementary file 1 [file mps-07-00020-s001.zip › mps-2859912-supplementary.pdf]

Supplementary Figure S1

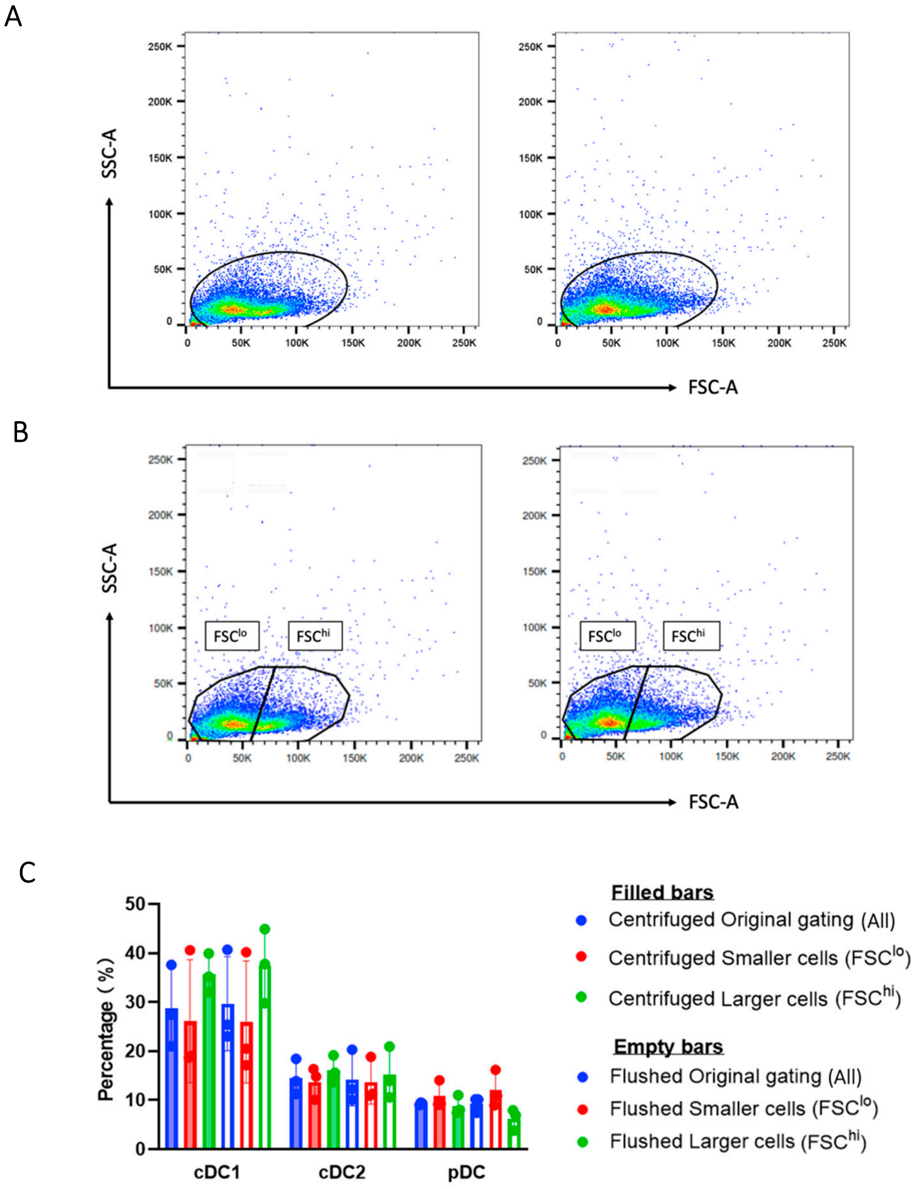

The proportions of DC subsets in the culture based on cell size. (A) The SSC-A/FSC-A gating including all cells in a wide gate for flushed cells (left) and centrifuged cells (right). A representative experimental day is shown. **(B)** Flushed cells (left) and centrifuged cells (right) from the same experimental day as (A), gated according to cell size (FSC-A). Cells smaller in size are gated as FSC^{lo} and larger cells as FSC^{hi}. **(C)** The proportions of different DC subsets based on FSC-A gating, comparing all cells (original gating from A, blue), smaller cells (FSC^{lo}, red), and larger cells (FSC^{hi}, green), for both bone marrow isolation methods (centrifuged or flushed).
